# Episodic Ataxias: Faux or Real?

**DOI:** 10.3390/ijms21186472

**Published:** 2020-09-05

**Authors:** Paola Giunti, Elide Mantuano, Marina Frontali

**Affiliations:** 1 Laboratory of Neurogenetics, Department of Molecular Neuroscience, UCL Institute of Neurology, London WC2N 5DU, UK; 2 Laboratory of Neurogenetics, Institute of Translational Pharmacology, National Research Council of Italy, 00133 Rome, Italy; elide.mantuano@ift.cnr.it

**Keywords:** episodic ataxia, channelopathies, *KCNA1*, *CACNA1A*, *SLC1A3*, *PRRT2*, *FGF14*, *SCN2A*, *SLCA1*

## Abstract

The term Episodic Ataxias (EA) was originally used for a few autosomal dominant diseases, characterized by attacks of cerebellar dysfunction of variable duration and frequency, often accompanied by other ictal and interictal signs. The original group subsequently grew to include other very rare EAs, frequently reported in single families, for some of which no responsible gene was found. The clinical spectrum of these diseases has been enormously amplified over time. In addition, episodes of ataxia have been described as phenotypic variants in the context of several different disorders. The whole group is somewhat confused, since a strong evidence linking the mutation to a given phenotype has not always been established. In this review we will collect and examine all instances of ataxia episodes reported so far, emphasizing those for which the pathophysiology and the clinical spectrum is best defined.

## 1. Introduction

Episodic Ataxias (EA) are a genetically heterogeneous group of autosomal dominant disorders characterized by attacks of movement incoordination (cerebellar ataxia) of variable duration and frequency, often accompanied by additional ictal and interictal symptoms. The two most frequent types are EA1 and 2, respectively produced by mutations of genes altering the function of K_v_1.1 and Ca_v_ 2.1 ion channel. Both diseases have a wide spectrum of clinical manifestations. Mutations of the *CACNA1A* gene, coding for the alpha subunit of Ca_v_ 2.1 channels, are the cause of two other allelic disorders, Spinocerebellar ataxia 6 (SCA6) and Familial Hemiplegic Migraine 1 (FHM1). These have features that can overlap with those of EA2. EA3 to 8 are either very rare or reported in single families. For only two of them, EA5 and EA6, the mutated gene is known, while for the others no responsible gene has been identified, except for EA8 possibly associated with two candidate genes (for references see below). In all of them, episodes of ataxia of variable frequency and duration are the most prominent and constant sign, even if sometime associated with other abnormalities (see below). EAs, however, should be distinguished from other genetic disorders in which episodes of ataxia are not the main clinical feature, but just a possible phenotypic variant.

There is not always strong evidence for the progressive increase in the number of EAs and for the expansion of their clinical spectrum (including both the group of EA1–8 and the disorders with a variant EA phenotype). Sometimes, the association between the gene mutation and the clinical picture is not confirmed by functional analyses, or other hypotheses are possible. In this review, we will describe the whole panorama of EAs, emphasizing those for which the pathophysiology and the clinical spectrum is best defined.

### 1.1. EA1 and EA2

EA1 and EA2 (Table 1) are due to mutations of the pore alpha subunit of an ion voltage-gated channel: K_v_1.1 coded by *KCNA1* and Ca_v_2.1 by *CACNA1A* gene respectively. They regulate K^+^ outflow or Ca^++^ influx across cell membrane in response to depolarization [1,2]. While the Ca_v_2.1 alpha subunit has a monomeric conformation, the K_v_1.1 pore of the channel complex has a tetrameric composition, rarely homo-oligomeric (four Kv1.1 subunits), and more often hetero-oligomeric (due to mix of Kv1.1 and K1.2 or K1.4 subunits) [3].

Six transmembrane segments, intracellular and extracellular linkers, and intracellular N- and COOH-tails form both alpha subunits [1,2]). Auxiliary subunits influence the channel expression and its biophysical properties [4,5]

Both channels are expressed in neurons and particularly in the cerebellum. K_v_1.1 is mainly expressed at the presynaptic terminals of cerebellar basket cells and in central and peripheral juxtanodal regions of almost all cerebellar myelinated axons [1]. Ca_v_ 2.1 are expressed in Purkinje and granule cells as well as in neuromuscular junctions [2]. In all cases the functional analyses of mutated channels showed a loss of channel function [1,2]. Functional analyses are usually performed either by introducing the mutated cDNA in Xenopus oocytes [6,7], or HEK293 cells [8,9], or by obtaining animal models [10,11], or directly on patient nerve excitability [12]. Mutations in both genes appear to alter channel-gating properties and neurotransmission, and to reduce channel expression [1,2]. (More details on the functional effect of EA1 and EA2 mutation are found in other papers of the present issue).

Both diseases have an early onset (infancy/childhood) and are characterized by attacks of vertigo, dizziness, movement incoordination triggered by physical exertion or emotional stresses [13,14], as well as by other stimuli like fever, or caffeine. However, the clinical phenotype of the two disorders can be differentiated: EA1 has short episodes lasting from seconds to minutes [1], while in EA2 the duration of attacks is longer (minutes to hours) [15]. Other ictal symptoms in EA1 are myokimia, spastic contractions, stiffening of the body, visual disturbances, and muscle cramps [1]; in EA2 they comprise visual abnormalities (diplopia, primary position nystagmus, oscillopsia), dystonia, and weaknes [13,15]. Interictal signs are also different: in EA1, almost all patients exhibit persistent myokimia/neuromyotonia, while the cerebellar function is typically normal. A persistent cerebellar ataxia, however, was reported in some patients [16]. In most EA2 patients, on the other hand, a persistent nystagmus and a mild vermian atrophy are present, and a permanent progressive cerebellar ataxia can develop over time [17].

The EA2 clinical picture is complicated by the possible overlap with other two autosomal dominant (AD) *CACNA1A* allelic disorders: Familial Hemiplegic Migraine type 1 (FHM1) and Spinocerebellar Ataxia type 6 (SCA6). FHM1, a migraine with a hemiplegic aura, is produced by gain-of-function mutations of the alpha subunit of Ca_v_ 2.1 channel [2]. SCA6 is an almost pure, progressive, neurodegenerative cerebellar ataxia due to the expansion of a polyglutamine repeat present in the COOH-tail of the alpha subunit [18,19]. The *CACNA1A* gene is bicistronic: the alpha subunit of Ca_v_ 2.1 channels is coded by the entire gene, while a transcription factor, containing a nuclear localization signal and a polyglutamine repeat, is coded from exon 40 to the 3′ end of the gene [20]. There is a remarkable clinical overlap between EA2, FHM1, and SCA6. Most patients with FHM1 have cerebellar signs and symptoms [2,21,22,23]. Over 50% of the EA2 patients have migraines [24]. Although SCA6 is characterized by a progressive ataxia, patients can present, at onset, with a fluctuating ataxia similar to EA2 [25,26,27]). On the other hand, some members of EA2 families with *CACNA1A* point mutations and no CAG expansion presented with prominent progressive ataxia reminiscent of SCA6 [28,29].

Epilepsy with or without developmental delay or permanent ataxia has also been frequently reported in the context of both EA1 [30,31,32,33,34,35]) and EA2 [36,37,38]. A severe form of Early Infantile Epileptic Encephalopathy (EIEE) is reported in some members of EA2 families with CACNA1A loss-of-function mutations [39,40,41]. These patients present with severe refractory seizures, starting in the first six months of life, global developmental delay evolving toward moderate to severe cognitive deficit, and variable motor symptoms (ataxia, tremors, spasticity, and alternating hemiplegia). The presence of the two phenotypes (EA2 and EIEE) within these families confirms that EIEE is part of the EA2 spectrum. De novo missense mutations of *CACNA1A* underlying EEIE cases have also been reported [42,43,44], mostly without a functional mutation analysis. Jiang et al. [38] performed a functional analysis of some de novo missense mutations causing EIEE which surprisingly revealed that some of them are loss- and other gain-of-function mutations. A severe epileptic phenotype was also present in two siblings with a heterozygous compound *CACNA1A* mutation (a missense and a 7 amino acid frameshift deletion), whose heterozygous relatives had only mild intellectual deficit, but no episodes of ataxia [45]. The phenotype of the two sibs also included features never previously reported in EIEE due to *CACNA1A* mutations, such as dysmorphisms and optic atrophy, leading to complete blindness. The unusual phenotype could be due to the combined mutations, but the presence of a chromosomal rearrangement in one of the parents, leading to a microdeletion in offspring, cannot be ruled out, as it would not be revealed by Whole Exome Sequencing.

Atypical phenotypes have also been reported in chromosomal microdeletion carriers, including part of (or the whole) *CACNA1A* sequence as well as other genes, but no assessment was made about the possible influence on phenotype of chromosomal imbalance, or of other gene deletions [39,46].

A non-progressive (or very slowly progressive) congenital ataxia with hemiplegic migraine and coma episodes is also due to de novo gain-of-function missense or indel CACNA1A mutations [36,47,48,49,50].

The spectrum of EA1 and EA2 phenotype has been progressively broadened, but the underlying mutation has not always been investigated through functional analyses, leaving some uncertainties about their effects, particularly when a novel/de novo mutation was found with a pathogenicity assessed only in silico. There is no doubt, however, that both EA1 and EA2 have an extremely variable clinical phenotype. There can be various reasons for this variability. It has been hypothesized that different mutations exert different loss-of-function effects or modify the function of the proteins that interact with the two alpha subunits [1,3,5,51]. Furthermore, the widely different clinical features in different members of the same family, sharing an identical genotype, [40,52,53] suggests the influence of modifiers in the genetic background. Moreover, clinical differences in monozygotic twins [54,55] suggest the influence of environmental factors. In any case, such a wide interfamilial and intrafamilial phenotypic variability makes it extremely difficult to define genotype-phenotype correlations [56].

The preventive management of both disorders mostly involves avoiding triggering stimuli that patients usually know quite well. Pharmacologically, little can be done to restore the channel loss-of-function. In EA2, however, acetazolamide (AAA)—a carbonic anhydrase inhibitor—can reduce the frequency of attacks, or completely abolish them [57]. So far, no placebo-controlled trials have been performed to assess the efficacy of AAA [58], and its therapeutic mechanism of action is not well understood. Magnetic resonance spectroscopy in EA2 patients showed increased pH in the cerebellum and cerebrum, which was corrected upon AAA administration [59]. Because of pH adjustments, channels and ionic conductance across neuronal membranes may be modulated, causing membrane hyperpolarization and a decrease in excitability, which can result in the observed reduction of attacks [1]. AAA, however, can have unwanted side effects such as kidney stones, hyperhidrosis, paresthesia, muscle stiffening with easy fatigability, and gastrointestinal disturbances [60], and it cannot always be prescribed.

In a pilot study and in an experimental trial, 4-Aminopyridine (4-AP)—a K^+^ channel blocker—was effective in reducing the frequency of EA2 episodes and improving patient wellbeing [61,62]. 4-AP acts on Purkinje cells, by ameliorating their pace-making activities [63,64].

In EA1, a number of drugs have been used with variable therapeutic effects [1,65]. AAA has been shown to be only occasionally effective in EA1 [66,67]. Considering the increased relative risk of seizures in these patients, various antiepileptic medications have been used with variable results, such as phenytoin [68,69,70], carbamazepine [8,32], and lamotrigine [16]. It should be noted, however, that over 50% of patients have never tried any preventive medication [16]. Very recently, small molecules selectively opening Kv1.1 channels have been investigated, thus offering a possible therapeutic drug for EA1 [71]. Moreover, in a recent preclinical study using a rat model of focal neocortical epilepsy gene, the overexpression of Kv1.1 was effective in controlling seizures [72], thus potentially offering the prospect for a targeted correction of the genetic defect in very severe EA1 cases.

### 1.2. EA5 and EA6 (Table 1)

EA5 was reported as affecting one in 71 analyzed EA families. The family carried a p.C104F missense mutation in *CACNB4* gene coding for the beta_4_ auxiliary subunit of Ca_v_2.1 channels [73]. The same mutation was also present in an unrelated family with a generalized epilepsy and no episodic ataxia [72]. Functional studies in vitro showed no alteration of channel kinetics, but the authors hypothesized other pathogenic effects not revealed by the analysis on channel gating [73]. This variant, although very rare, is present in the general population (Genome Aggregation Database and Ensembl). It is well known that *CACNB4* mutations are causing different types of epilepsy [65]. No other cases presenting with episodes of ataxia, however, have been so far reported, despite the inclusion of *CACNB4* gene among those screened for mutations in EA patients [74,75]. Jen and Wan [13] have called into question whether there are sufficient data to support the designation of EA5.

EA6 patients carry mutations in the excitatory amino acid transporter 1 (EAAT1), a Na^+^-dependent glutamate transporter coded by gene *SLC1A3* and expressed in cerebellar astrocytes known as Bergmann glia [75,76,77,78]. The patients present with EA as well as progressive ataxia, seizures, and migraine headaches with [76] or without [77] prolonged alternating hemiplegia, triggered by head trauma or fever. The functional study of the mutations showed a loss of transporter function with impaired glutamate uptake. EAAT1, however, also has a function as ion channel; the same mutation p.P290R reported by Jen et al. [76] was found to induce a gain of this second type of function. It has been proposed that a similar mechanism is the primary cause of EA6, rather than the reduction of glutamate uptake [79,80]. A recent variant of EAAT1 carried by a patient presenting with a severe migraneous headache was shown to impair K+ binding to the mutant protein [81], thus introducing a novel mechanism causing glutamate transport dysfunction.

### 1.3. EA 3,4,7,8 (Table 1)

Each of these EAs, all AD, has been reported in one or two large families presenting with a phenotype largely overlapping with that of EA1 or EA2, except for a few clinical signs. EA3 had short attacks with ictal and interictal signs similar to EA1 except for vertigo and tinnitus [82]. EA4 was similar to EA2 except for a late onset [83]. EA7 was similar to EA2 except for absence of interictal nystagmus [85]. Attacks in EA8 patients were similar to EA2, but were not sensitive to AAA, while responding to clonazepam [86].

For EA3 and EA4, linkage analysis excluded *KCNA1* and *CACNA1A* as possible mutation sites [82,84].

A genome-wide linkage analysis in the EA3 family reported by Steckley et al. [82] mapped the gene in a 4-cM region on 1q42 with a relatively high lod score. However, this result was obtained only after adapting the linkage parameters to the family. This was done by considering three affected patients that did not carry the putative haplotype as phenocopies, and by considering five healthy family members with the disease haplotype as instances of incomplete penetrance. Otherwise, the lod score would have been below the significance cutoff [87].

In a 91-year-old patient of the EA4 family [83], a neuropathological brain examination showed, among other abnormalities, the presence of poliglutamine repeats in Purkinje and granule cells, without intranuclear inclusions, similar to those of SCA6 brains [88].

EA7 was mapped in a 10-centimorgan on chromosome19q13 with a lod score slightly above the significance cutoff [85]. Sequencing of two candidate genes *KCNC3* and *SLC17A*7, lying in this region, did not reveal any mutation. So far, no other EA3, EA4, or EA7 families have been reported, making it possible that gene mapping results are not sufficiently reliable, or that the disorder was, in fact, one of the known types of fluctuating ataxia, such as SCA6.

EA8 was mapped in a relatively large region of 18.5 Mb on chr. 1p36.13-p34.3 with a lod score very near to cutoff [86]. Exome sequencing in the latter region revealed a variant in two genes *SPG2* and *UBR4*, the pathogenicity of which was predicted in silico to be stronger in the second gene than in the first. *UBR4* is an ubiquitin ligase protein that is interacting with calmodulin and co-localizes with ITPR1 with a potential for disrupted Ca^++^ control within neuronal cells [86]. Two additional EA cases with a UBR4 mutation have been reported [75], but, as in the previous case, no functional analysis was performed.

### 1.4. Episodiac Ataxias in the Context of Other Disorders

Some of EA-like phenotypes have been reported in the context of other genetic disorders, either as the most prominent feature or in association with other abnormalities. This could explain, at least in part, the presence of patients with typical EA1 or EA2 clinical picture who do not carry mutations in the respective genes [16,17,74,89]. Table 2 shows an updated list of these disorders. Some of them are related to mutations in genes coding for ion channels (*SCNA2. KCNA2, KCND3, NALCN*) or ion pumps (*ATP1A2* and *ATP1A3*), or proteins interacting with ion channels (*PRRT2* and *FGF14*) or Na^+^-dependent transporters (*SLC2A1*). For most disorders in this group, the EA variant phenotype is reported in a single case, and often associated with other abnormalities (see references in Table 2). For *PRRT2* and *FGF14*, and *SCNA2*, instead, the inclusion of EA in the disease spectrum was confirmed in several patients.

#### 1.4.1. PRRT2

*PRRT2* (Proline-rich Transmembrane protein 2) mutations underlie three major phenotypes: Benign Familial Infantile Epilepsy, Paroxysmal Kinesigenic Dyskinesia with or without infantile convulsions, and Infantile Convulsions with Choreoathetosis. About 5% of patients bearing *PRRT2* mutations display other disorders such as episodic ataxia, hemiplegic migraine, developmental delay, and intellectual disability [99,100,101,102]. The pleiotropy associated with *PRRT2* mutations is not related to any specific genotype-phenotype correlation, as most mutations are loss-of-function [103]. PRRT2 is a neuron synaptic protein expressed at the highest levels in cerebellum, basal ganglia, and neocortex. At the nerve terminal, PRRT2 endows synaptic vesicle exocytosis with Ca^++^ sensitivity by interacting with Ca^++^-sensing machinery, thus playing an important role in calcium triggered exocytosis [103,104,105]. *PRRT2* knock-out mice show heightened spontaneous and evoked activity at the network level associated with increased excitability of excitatory neurons [105]. PRRT2 was also found to interact with Na^+^ channels and their direct consequences on neuronal excitability [106]. Carbamazepine is reported to be an effective drug for *PRRT2* mutations [99,102].

#### 1.4.2. FGF14

Mutations in *FGF14* gene, coding for Fibroblast Growth Factor 14, underlie SCA27, a late onset, slowly progressing cerebellar ataxia with extrapyramidal features such as postural tremor, head titubation, and parkinsonism [107,108]. This gene, highly expressed in the brain and especially in granule and Purkinje cells, controls channel gating and axonal targeting of Na_v_ 1.1, 1.2 and 1.6 channels [109,110]. It also regulates Ca_v_2.1 channels [111] and is required for Purkinje cell spontaneous firing [112]. Several patients have been recently reported with an autosomal dominant episodic ataxia harboring *FGF14* mutation [74,113,114,115,116,117]). Episodes have variable onset, frequency, and duration and are characterized by vertigo, dizziness, and unsteadiness often triggered by fever. Tremor and nystagmus are usually interictal signs. Responsiveness to AAA has been reported [116,117]). Piarroux et al. [117] suggest to consider FGF14-related episodic ataxia as Episodic Ataxia type 9.

#### 1.4.3. SCNA2

*SCN2A* encodes the alpha subunit of the voltage gated neuronal Na_V_1.2 channel. Loss-of-function mutations are associated with a severe form of epilepsy, and/or intellectual disability, and autistic traits. Gain-of-function pathogenic variants of SCN2A underlie the Benign Familial Infantile-Neonatal Seizure (BFNIS) affecting children before three months of age and disappearing with age. Seizures in BFNIS can be controlled with Na^+^-channel blockers such as phenytoin and carbamazepine, while they are ineffective for the severe form [118,119]. Some BFNIS patients present with EA either co-occurring with epileptic spells [120,121,122,123,124,125] or as the only clinical feature [124,125,126]. EA does not respond to Na^+^ channel blockers, suggesting a distinct pathophysiological mechanism, which, however, has not been identified so far. Cases of EA responding to AAA has been reported [123,126].

#### 1.4.4. Mitochondrial Disorders

Another group of diseases, for which single EA cases have been reported, is related to mitochondrial metabolism (*PDHA1*, *TPK1*, and *DARS2* gene). A common trait of these diseases is the presence of CSF and plasma lactic acidosis, never found in EA1–8. In these cases, EA does not present as the prominent symptom, but is associated with other abnormalities.

#### 1.4.5. Other Disorders

In a final group of highly heterogeneous disorders, the only disease with a prominent EA phenotype is GLUT1 deficiency syndrome, due to mutations in *SLC2A1* gene on chromosome 1p34. The gene codes for the membrane protein responsible for glucose transport across the blood-brain barrier, maintaining the continuous high glucose and energy demands of the brain [127]. Mutations in this gene cause the Glucose Transporter 1 deficiency syndrome (GLUT1 DS) and reduce the maximum velocity of glucose uptake [128,129]. The phenotype is characterized by early onset epilepsy, mild to severe developmental delay, and acquired microcephaly. Hypoglycorrhachia and low CSF lactate are diagnostic symptoms. Epileptic attacks and other manifestations, such as developmental delay and intellectual disability, are often controlled by a ketogenic diet. Ketone bodies diffusing across the blood–brain barrier, facilitated by a monocarboxylic acid transporter, serve as an alternative energy source for brain metabolism [128]. About 10% of *SLC2A1* mutation carriers do not have the classic epileptic phenotype and present with paroxysmal exertion-induced dyskinesia which, in some cases, is reported as EA [129]. Often patients presenting with EA also have other neurological abnormalities [130,131,132,133,134], but cases with an almost pure EA have been reported [129,135]. AAA in these cases controls ataxic spells [129,132,135].

## 2. Conclusions

EAs are a group of disorders with many complex aspects. A first complicating element is the high variability of phenotypes associated with different mutations in the same gene. Some of these could be related to the mutation functional effect (gain- or loss-of-function), as e.g., for CACNA1A or SCNA2 gene, but even within the same functional effect the phenotypes are highly variable. The site of the protein mutation can rarely predict the phenotype, as in EA1 patients carrying a p.T226R substitution who have quite different clinical features from those carrying p.T226A or p.Thr226M mutations [1]. Moreover, phenotypic variability was also reported among family members sharing an identical mutation [52,53,54] or in monozygotic twins [54]. The underlying genetic and pathophysiological basis of the extreme variability within the same disease has not been clarified, thus preventing any genotype/phenotype correlation. Genetic modifiers as well as environmental factors are likely to influence the clinical picture, but both have been poorly investigated so far. The recent possibility of analyzing channel function in iPSC derived neurons from patient fibroblast [136,137]—hence sharing the patient genetic background—could perhaps throw some light on this problem.

In some disorders, the broad extension of the clinical spectrum is documented in reports of single cases with an atypical phenotype. This leaves room for doubts about their real association with the reported mutation particularly in de novo cases—for which genotype/phenotype co-segregation cannot be assessed—and/or when gene variants are the result of NGS methods. The limits of these methods may hinder other mutations in sites not sufficiently covered or undetectable (e.g., repeat expansion, microdeletion). Moreover, more uncertainties are caused by the fact that in these cases no functional study of the mutated protein is usually performed.

In conclusion, it would be helpful to reconsider the whole nosography of EAs in the light of the present and future genetic and pathophysiological evidence.

## Figures and Tables

**Table 1:** List of Episodic Ataxias (EA1–EA8), all with an autosomal dominant inheritance.

Disease	OMIM	Gene/MAP	Phenotype	Ref.
Episodic Ataxia 1 (EA1)	160120	*KCNA1*	Early onset, short attacks (sec to min), myokimias, cramps, spastic contraction	[1,16]
Episodic Ataxia 2 (EA2)	108500	*CACNA1A*	Early onset, long attacks (min to hours), interictal nystagmus	[2,13,15,17]
Episodic Ataxia 3 (EA3) Single family	606554	Linkage excluded EA1 & 2 Gene maps 1q42?	Similar to EA1 except for presence of tinnitus	[82]
Episodic Ataxia 4 (EA4)	606552	Linkage excluded EA1 & 2	Similar to EA2 except for absence of interictal nystagmus	[83,84]
Episodic Ataxia 5 (EA5) Single family	613855	*CACNB4*??	Detailed phenotype not reported	[73]
Episodic Ataxia 6 (EA6)	612656	*SLC1A3*	Ataxia episodes, migraine, seizures, alternating hemiplegia	[76,77,78,79,80,81]
Episodic Ataxia 7 (EA7) Single family	611907	Maps 19q13	Similar to EA2, except for interictal nystagmus	[85]
Episodic Ataxia 8 (EA8) Single family	616055	*UBR4??* Maps 1p36.13-p34.3	Myokimias, persistent intention tremor	[86]

?? indicate uncertainties described in the text.

**Table 2:** List of disorders that can include episodic ataxia as a phenotypic variant of complex phenotypes.

DISEASE	OMIM	GENE	PHENOTYPE ^1^	INHERITANCE ^2^
Disorders Due to Mutations in Ion Channels, Pumps or Transporters				
Hypotonia, infantile, with psychomotor retardation and characteristic facies 1 Congenital contractures of the limbs and face, hypotonia, and developmental delay	616266	*NALCN*	Dysmorphisms, mental retardation, seizures [90]	AD
SCA19/22	607346	*KCND3*	Slowly progressive cerebellar ataxia, vertigo, dysarthria, earfulness [75]	AD
EIEE 32	616366	*KCNA2*	Uncontrolled seizures, developmental slowing or regression [91]	AD
Bening Familial Neonatal-Infantile Seizure ^3^	607745	*SCN2A* ^3^	Clusters of afebrile seizures with onset within the first 3 months, without neurologic sequelae	AD
Cerebellar Ataxia, Areflexia, Pes cavus, Optic Atropy, Sensorineural hearing loss (CAPOS)	601338	*ATP1A3*	Cerebellar ataxia, areflexia, pes cavus, Optic atrophy, and hearing loss [92]	AD
Episodic Kinesigenic Dyskinesia 1 ^3^	128200	*PRRT2* ^3^	Attacks of dystonic and/or choreic and/or athetosic movements	*AD*
SCA 27 ^3^	609307	*FGF14* ^3^	Postural tremor, slowly progressive ataxia, and cognitive deficits	AD
Mitochondrial Disorders				
Pyruvate dehydrogenase E1-deficiency	312170	P*DHA1*	Dysmorphisms, developmental delay, cerebral atrophy, lactic acidosis [93]	X-LD
Episodic Encephalopathy type 5 (THMD5)	614458	*TPK1*	Encephalopathic episodes with increased CSF and serum lactate [94]	AR
Leukoencephalopathy w. brainstem & Spinal Cord involvement & Lactate elevation (LBSL)	611105	*DARS2*	Delayed development, spasticity, neuropathy, leukoencephalopathy, lactic acidosis [95]	AR
Others				
GLUT1Deficiency Syndrome 1 ^3^	606777	*SLC2A1* ^3^	Developmental delay, epileptic encephalopathy, spasticity, low CFS glucose and lactate	AD
Schuurs-Hoeijmakers Syndrome	615009	*PACS1*	Dysmorphisms, developmental delay, congenital heart disease [96]	AD
Citrullinemia, Hypomorphic	215700	*ASS1*	Developmental delay, seizures, hyper- ammoniemia. hypercitrullinemia [97]	AR
SCA35	613908	*TGM6*	limb and gait ataxia, hyperreflexia, dysarthria, hand tremor [5]	AD
Leber Cong.Amaurosis X;	611755	*CEP290*	Allelic disorders with very different	AR
Joubert Syndr. 5	6101886		phenotypes involving retinal dystrophy	
Meckel Syndrom 4	1113461		cerebellar atrophy; encephalocele and other	
Bardet-Biedl Syndr. 14	5991610		malformations; obesity and mental	
Senior-Loken syndrome 6.	189		retardation; retinal degeneration [98]	

^1^ Main phenotypic features are included and references reporting EA in each context, except for a few disorders described in detail in the text. ^2^ X-LD X-linked dominant, AD—autosomal dominant, AR—autosomal recessive. ^3^ references reported in the text.

## Abbreviations

AAA	Acetazolamide
AD	Autosomal Dominant
AR	Autosomal Recessive
BFNIS	Benign Familial Neonatal-Infantile Seizure
CSF	Cerebro-Spinal Fluid
EA	Episodic Ataxia
EIEE	Early Infantile Epileptic Encephalopathy
FHM	Familial Hemiplegic Migraine
GLUT1DS	Glucose transporter 1 deficiency syndrome
NGS	New Generation Sequencing
SCA	Spino-cerebellar Ataxia
X-LD	X-Linked Dominant
4-AP	4-aminopyridine
